# Protective Policy Index (PPI) global dataset of origins and stringency of COVID 19 mitigation policies

**DOI:** 10.1038/s41597-022-01437-9

**Published:** 2022-06-16

**Authors:** Olga Shvetsova, Andrei Zhirnov, Abdul Basit Adeel, Mert Can Bayar, Onsel Gurel Bayrali, Michael Catalano, Olivia Catalano, Hyoungrohk Chu, Frank Giannelli, Ezgi Muftuoglu, Dina Rosenberg, Didem Seyis, Bradley Skopyk, Julie VanDusky-Allen, Tianyi Zhao

**Affiliations:** 1 Binghamton University, Binghamton, New York USA; 2 University of Exeter, Exeter, Devon UK; 3 Penn State University, State College, Pennsylvania USA; 4 MS-MPH, unaffiliated researcher, Binghamton, New York, USA; 5 Rutgers, the State University of New Jersey, Piscataway, New Jersey USA; 6 National Research University-Higher School of Economics, Moscow, Russia; 7 Boise State University, Boise, Idaho USA

**Keywords:** Government, Politics

## Abstract

We have developed and made accessible for multidisciplinary audience a unique global dataset of the behavior of political actors during the COVID-19 pandemic as measured by their policy-making efforts to protect their publics. The dataset presents consistently coded cross-national data at subnational and national levels on the daily level of stringency of public health policies by level of government overall and within specific policy categories, and reports branches of government that adopted these policies. The data on these public mandates of protective behaviors is collected from media announcements and government publications. The dataset allows comparisons of governments’ policy efforts and timing across the world and can serve as a source of information on policy determinants of pandemic outcomes–both societal and possibly medical.

## Abstract (summary)

**Table:** 

Measurement(s)	institutions, levels of government, dates and stringency of COVID-19 public health policy announcements
Technology Type(s)	content search of media and governement sources, expert coding and classification
Factor Type(s)	government office • government level • time in the pandemic • country • region
Sample Characteristic - Organism	government-announced COVID-19 public health policies
Sample Characteristic - Environment	regions, also aggregated to countries
Sample Characteristic - Location	global

## Background & Summary

The onset of the COVID-19 pandemic in 2020 constituted a unique event when a novel health threat arose simultaneously across global communities and elicited responses from political authorities, globally. Even though eventual peaks of infection occurred at different times in different countries, the threat of a public health disaster became apparent to the governments in all countries at about the same time. Non-medical interventions (NMI) were the primary method to reduce the spread of infection during the entire first year of the pandemic. Despite the well-justified expectation that the scientific community would develop a vaccine and remedy for this virus, mass vaccination did not begin until the end of 2020 and early 2021. Until then, the public health solutions to the crisis were thus limited to behavioral measures such as mask-wearing mandates, social distancing, and closure of borders. It was through political process that government officials everywhere introduced protective public health policies ranging from instituting lockdowns to requiring the wearing of face covers to alter behavior. While these interventions were epidemiologically sound based on historical experience, their direct immediate benefits to individuals were difficult to measure, whilst their social and economic costs were immediately apparent. The pandemic thus presented the political incumbents worldwide with a *choice situation* in terms of what, if any, public health measures they would enact. This dataset documents the policy choices that various governments worldwide have made vis-à-vis a shared public health threat.

While medical practitioners and scholars of public health and public policy around the world are still taking stock of these decisions, they need reliable and cross-nationally comparable data on the relative performance of their governments –attributing policy measures to specific levels and branches–in protecting their publics in a health crisis through adequate policy measures. This assessment of the relative efficacy of different governments is an urgent task, since repeated waves of the current pandemic are possible, and other medical crises are likely in the future.

The core of the pre-vaccine response to the pandemic has been a mitigation strategy which sought to alter individual behaviors through government imposed restrictions. Policy activity was informed by previous responses to respiratory infections, such as influenza and SARS-CoV (2003). The purpose of the construction of this dataset is to describe different government responses and quantify the strength of their political effort to distance people to prevent the spread of a respiratory based illness. This dataset documents the stringency of policies in fifteen public health categories by the level of government (i.e., national or subnational) and government branch (i.e., executive, legislative, judicial, bureaucracy) announcing those policies. These records are based on the original data of announced policy measures, collected for each subdivision of the included countries at the ISO 3166-2 standard level^1^. The datasets contain information about 81 countries in North America, Central America, South America, Europe, the Middle East, and Asia, with the records from January 1, 2020 and at least through April 30, 2020, but for most countries extending through the summer or until the end of 2020.

In addition to providing the stringency of public health policies by category, we construct Protective Policy Indices (PPI) to measure the aggregate policy stringency in each included subnational unit. We produce separate indices based on the policy efforts of national and subnational governments (National PPI and Regional PPI respectively), as well as their combined policy output (Total PPI). We use two methods (methods 1 and 2 as described below) for calculating Protective Policy Indices because the understanding of what was required in COVID-19 mitigation changed significantly by the end of the spring of 2020. For example, the World Health Organization (WHO) initially recommended against mask-wearing mandates in the early stages of the pandemic but then formally recommended wearing masks on June 5, 2020^2,3^. Method 1 reflects that initial understanding, and method 2 reflects a more recent view of effective mitigation. Both methods are outlined in the Method section below. We calculate PPI values using both methods for the entire duration of data coverage in order to maintain consistency in the data and leave it for the users to decide if they want to alter the method at any date in 2020 in their analyses.

Beyond the records of subnational units, we also produce country-level calculated data which include the averages across the subnational values of policy stringency weighed according to the units’ population shares. Furthermore, we include a file that reports the episodes of policy-making, national and subnational, and includes information on the branch of government initiating the policy change as well as the differential in policy stringency in each category that results from that policy change.

The theoretical focus of our project is on the institutional origins of the policies, and we have sought to investigate which parts of the government and which political processes contributed to the formation of the COVID-19 policy response, how the political incumbent’s position in the structure of government affected their ability and willingness to make policies of a varying degree of stringency. Accordingly, the present dataset both locates the policy measures in space and time and records their origins in the governmental structures. Since our focus is on the policy effort rather than its effects, and limited to policy-making, rather than implementation, we do not look into enforcement and compliance. Our data can, however, be matched with, e.g., mobility data, in order to estimate the latter^4^.

These data will contribute to the research on the political and non-political determinants of public health policies and will help to assess the effects of timely public health response for the health outcomes. Our perspective and theoretical goal is to be able to comparatively assess the behavior of politicians in office as they found themselves responsible for the NMI. While the data reflects the stringency of NMIs and thus can serve as one of the inputs to medical efficacy research, the theoretical drive for data collection was to compare regimes and political systems according to their capacity and willingness to respond to a crisis.

The data were partially used in research on the political determinants of COVID-19 mitigation strategies, on public opinion determinants in pandemic, on compliance behavior, and on variation in pandemic outcomes^5–10^. The dataset consistently codes governments’ policy efforts across the globe and specifies, explicitly, their institutional origin, viz. the inclusion of the level and branch of government that produces the policy. The main advantage of these data for analyzing the consequences of public health policies is to provide a complex continuous measure of the public health regime of each government.

To facilitate the exploration of our data by other researchers and interested parties, we built an ArcGIS Dashboard, which is available at https://elcamaleon.binghamton.edu/portal/apps/opsdashboard/index.html#/cc61a3652eb74b8ea8864928e8026aa1. This dashboard offers cartographic, textual, and graphical visualizations of daily PPI data at national and subnational levels. For comparative purposes, a separate map layer visualizes daily case rates, using data from the Center for Systems Science and Engineering at Johns Hopkins University^11^. The dashboard allows users to explore the temporal and spatial dynamics of global PPI by means of A) selecting map features at the national level (polygons) or subnational level (points), B) toggling map layers, and C) filtering data using the date selector.

## Methods

### Categories of public health policies

We identify and code policies falling into several categories: border closures (international and domestic), school closures, social gathering and social distancing limitations, home-bound policies (curfew, stay-at-home, lockdown), medical isolation policies (self-isolation and mandatory quarantine), closure/restriction of businesses and services (closure of nonessential businesses, restaurants, entertainment venues, government offices, public transportation, work from home requirements), the introduction of the state of emergency, and requiring mandatory personal protection equipment and physical distancing.

The border closures categories were of paramount importance in responding during the ‘alert period’ (the very first weeks) of the pandemic and remained important in preventing new infections due to inter-territorial externalities in public health response. The relative weight of border closures in the PPI is the main difference between Methods 1 and 2.

Limits/closures on schools, large venues, restaurants, non-essential businesses, public transportation closures, government offices, and places of employment are policies aimed at reducing infections from community spread. Each of these is also graduated based on the strictness of the restriction implemented. We code them separately because different governments chose to close some but not others and/or implemented closures of each at different times.

Restrictions on mobility for the healthy population (personal mobility, social gatherings) are similarly aimed to reduce infections from community spread. Again, these categories are graduated based on the stringency of the measures put in place. These measures are distinct from the previous set because they represent an infringement on individual liberties. They do, however, function similarly from an epidemiological perspective to limit community spread.

Publicly mandated hygiene practices-mandatory PPE and quarantine for suspected exposures - are intended to minimize/prevent community spread. This works by severely limiting the ability of the virus to spread through droplets expelled by individuals who are infected but might not know in the case of PPE, and by physically isolating the carrier from the rest of the population in the case of quarantine. Note that these do not include changing requirements imposed on medical facilities during the pandemic since we only measure public-health policies and not medical practices.

Lastly, state of emergency is a category that captures the commitment of the government to handling the crisis through policy. It enables the adoption of some policies that may violate civil liberties and enables enforcement of many of the hygiene practices and business restrictions.

We differentiate between the policies made by national and subnational governments. For each category of public health policies, subnational unit, and day, we produce three values: the stringency of policies made in that category by the subnational government, the stringency of policies made in that category by the national government, and the total stringency of policies in that category, computed as the highest between the national and subnational values. Often policies announced by different level governments were duplicates of one another at a given time. For example, there may be a nationally-mandated self-isolation period and a self-isolation/quarantine requirement for a region based on the same or similar criteria. In this case, the national policy would be counted towards the value of the National PPI and the subnational policy towards the Regional PPI. The policy would take the highest value out of the two for the value that goes into calculating the Total PPI (see Appendix A3).

Note that there is variation on stringency within the policy categories, with some policy adoptions being more stringent than others (i.e. self-isolation versus lockdowns, partial school closings versus full school closings). To this end, we weighed more stringent policies in each category in the index more heavily. These values are normalized to range between 0 and 1. Data are collected from national and sub-national news sources, government resources, and press releases. The auxiliary file “changes_regions_m1.csv” contains a column with the source links for each recorded change in the policy variables. The changes reported in this file are what gives rise to the running values of the policy variables and their aggregates in the main files.

Because all categories that we code are public mandates for mitigation, sources include official documents, news reports, as well as the announcements of relevant policies made by the officials. To match the content of policies with specific policy categories and maintain comparability across countries, the coders were trained to use the meaning rather than the language of the policy. Appendix 2 describes the meaning of policies as coded. Global regions curators regularly met to discuss the challenges of interpretation that their teams encountered in specific countries.

### Index calculations

We calculate the Public Health *Protective Policy Index* (PPI) based on coded public health policy responses. The dataset contains three types of PPI scores: regional PPI for each subnational unit on each day; national PPI for each subnational unit on each day, based on nationally issued policies; and Total PPI for each subnational unit on each day. The Total PPI reflects the strictest between the national and subnational policies adopted within each category for that unit for that day. The indices are scaled to range between 0 and 1.

We seek to weigh public health policies according to their expected efficacy to construct the index of these policies. Under normal circumstances, in recurrent crises, previous outbreaks involving similar pathogens have enabled evaluation of the effectiveness of such policies based on observed transmission within and between communities. These responses are pre-considered to the extent possible though they generally focus on influenza^12–14^. Due to the unique virulence of the coronavirus responsible for COVID-19, the public-health community’s initial perception of what constituted a stringent response was based very much on the evolving expectations of the disease’s unique features based on historical experience with related outbreaks (such as MERS and SARS) (see CDC, 2020 pp. 554 for a discussion of uncertainty around transmission during the early months of the pandemic).

We use two methods for computing indices to account for the evolving understanding of the most effective measures to inhibit the spread of the coronavirus. The first method of computing the PPI (**indexing method 1**), as shown in Table 1 reflects what was known in early- to mid-spring 2020. Note that these weights were deemed appropriate during the alert phase (the very first days/weeks of rising global infections) and the early weeks of the pandemic phase. The second method (**indexing method 2**, shown in Table 2) reflects the changes in guidance later in the year. All such judgements and any such index construction could be at best based on evidence at the time of index construction, and the initial expectations of the stringency were in fact derived from experiences with prior, non COVID-19, pandemics and epidemics. The approach here is that at the beginning and even today, the weight was based on the prevailing idea that the more isolating the measure the stronger it is. So the more strictly the measure isolated someone, the heavier the weight.

**Table 1:** Weights of categories in PPI, method 1.

Category	Variable	Weight in PPI
1. International and domestic air borders closure	borders.air_bord	0.075
2. International and domestic land borders closure	borders.land_bord	0.075
3. International and domestic sea borders closure	borders.sea_bord	0.075
4. Limits on size of social gatherings	soc_and_schls.soc_gath	0.1
5. Closing of schools	soc_and_schls.schools	0.1
6. State of emergency	emerg.all	0.075
7. Closure of entertainment venues /stadiums	places.venues	0.025
8. Closure of restaurants	places.restrts	0.05
9. Closure of non-essential businesses	places.ne_busn	0.05
10. Closure of government offices	places.gov_offs	0.05
11. Working from home requirement for businesses/organizations	places.wfh	0.025
12. Personal mobility restrictions	ind_locat.ind_mob	0.125
13. Self-isolation and/or quarantine requirements	ind_locat.med_stay	0.075
14. Public transportation closures	ind_locat.publ_tr	0.05
15. Mandatory wearing of PPE/ masks	masks.all	0.05

**Table 2:** Weights of categories in PPI, method 2.

Category	Variable	Weight in PPI
1. International and domestic borders closure	borders.all	0.110
2. Limits on size of social gatherings	soc_and_schls.soc_gath	0.110
3. Closing of schools	soc_and_schls.schools	0.110
4. State of emergency	emerg.all	0.041
5. Closure of entertainment venues /stadiums	places.venues	0.027
6. Closure of restaurants	places.restrts	0.055
7. Closure of non-essential businesses	places.ne_busn	0.055
8. Closure of government offices	places.gov_offs	0.055
9. Personal mobility restrictions	ind_locat.ind_mob	0.137
10. Self-isolation and/or quarantine requirements	ind_locat.med_stay	0.027
11. Quarantine	ind_locat.med_quar	0.055
12. Working from home requirement for businesses/organizations	places.wfh	0.027
13. Public transportation closures	ind_locat.pub_transp	0.055
14. Mandatory wearing of PPE/ masks	med_mandate.masks	0.082
15. Personal distancing rules 1.5–2 m	med_mandate.dist_mand	0.055

Because the efficacy of measures in pandemics/epidemics/infectious disease management etc., depends on the virus, and even that varies with its variants, we make no claim that the weights in our indices are unique and *accurately* assigned. We believe that they are *reasonably* assigned. Future will tell, and for that purpose, our codebook provides all components as well as their individual constituent policies in order for researchers to have easy access to reassigning the weights. The codebook contains formulae for calculating full PPIs and category stringencies for types of policies and for all calculated variables. All indices thus can be adjusted to recalculate as needed, from the input–coded variables–to match the nature of research questions that the users might pursue or the future evidence of the relative efficacy of these measures.

At the country level, the Average Regional PPI, Average National PPI, and Average Total PPI are computed by weighing the different units’ PPI values by the units’ population shares (see Appendix A3).

### Data collection and processing workflow

**Fig. 1:**
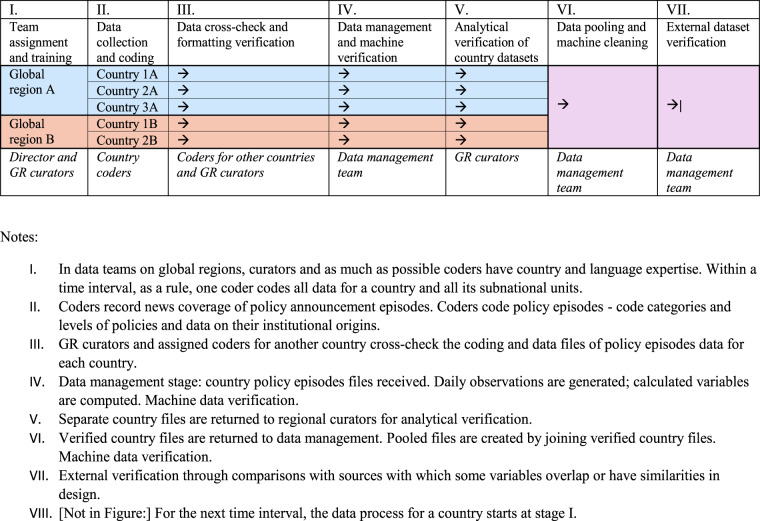
Data process for each time interval. This figure presents data collection and processing workflow schema used during each of the time intervals for which the data was collected.

As described in Fig. 1, the data collection process consists of seven phases for each time interval under the collection. Three time periods were established as intervals for data collection, within each of those the full process in Fig. 1 applies: the first period was between January 1 and April 24, 2020; the second period lasted between April, 24 and July, 31, 2020; the third interval covered the period from August 1, 2020 until the end of the year. The coder or coders for individual countries and their sub-national units remained consistent within each time period. Coders worked as teams based on global regional (GR) grouping. Senior personnel serving as GR curators generally remained consistent for the entirety of the project.

## Data Records

We have created a Github repository (https://github.com/COVID-policy-response-lab/PPI-data) to store the datasets with the Public Health Protective Policy Index and its components. A copy of the included datafiles, as described below, was deposited with openICPSR^15^. It presently requires creating an account with the depository. Data access is free. Data location is at https://www.openicpsr.org/openicpsr/project/123401. The datasets are stored as csv files with five types of layouts.

**“PPI_country_m1.csv”** is a file with country-level aggregates of region-level PPIs, computed using method 1, and their components. Each row corresponds to a country-date. The rows are identified using the country name (cname), numeric and 2-letter ISO 3166-1 codes (isocode and isoabbr respectively), as well as a date variable.

The names of the policy variables contain four components: the name of the broader category, the name of the category, the level of issuing government (“nat” refers to the national policies, “reg” refers to the subnational policies, and “tot” refers to the combination of national and subnational policies), as well as suffix “ave”. For example, the average Total PPI is denoted as “ppi.all.tot.ave”, and the average stringency of the closures of air borders by the national government is denoted as “borders.air_bord.nat.ave”. See the codebook for the complete list of variables.

**“PPI_country_m2.csv”** is a file with country-level aggregates of region-level PPIs, computed using method 2, and their components. The identifying variables and the naming convention for the policy variables is the same as in “PPI_country_m1.csv”, with the addition of suffix “0.2” at the end of the policy variable names.

**“PPI_regions_XX_m1.csv”** (replace XX with the 2-letter ISO 3166-1 country codes) are country-specific files with region-specific PPIs, computed using method 1, and their components. The identifying variables include the numeric and 2-letter ISO 3166-1 codes of the country (isocode and isoabbr respectively), the name of the region (state_province), its ISO 3166-2 code (iso_state), as well as a date variable. The names of the policy variables contain three components: the name of the broader category, the name of the category, and the level of issuing government (“nat” refers to the national policies, “reg” refers to the subnational policies, and “tot” refers to the combination of national and subnational policies). For example, the average Total PPI is denoted as “ppi.all.tot”, and the stringency of the closures of air borders by the national government is denoted as “borders.air_bord.nat”.

**“PPI_regions_XX_m2.csv”** (replace XX with the 2-letter ISO 3166-1 country codes are country-specific files with region-specific PPIs, computed using method 2, and their components. The identifying variables and the naming convention for the policy variables is the same as in “PPI_regions_XX_m1.csv”, with the addition of the suffix “0.2” at the end of the policy variable names.

**“changes_regions_m1.csv”** is an auxiliary file that describes the changes in the policy states, as recorded in the “PPI_regions_XX_m1.csv” files. Each row in this file corresponds to a change in a value of a policy state variable in a region and of a specific government level. The case identifying variables include the name of the country (cname), the numeric and 2-letter ISO 3166-1 code of the country (isocode and isoabbr, respectively), the name of the region (state_province) and its ISO 3166-2 code, date, policy dimension, and a marker of policies issued by a regional government (subnational). Among others, the attributes included in this file include the branch of the government (branch) and the date when the change was announced (report_date).

## Technical Validation

### Data verification

The data collection process included a number of stages at which the coding of policies was verified to ensure internal consistency of coding.

1. After the individual coders submit spreadsheets with their coding to the global regions (GR) curators (see Fig. 1), the GR curators verify sources and coding (either personally or by assigning a country data verification to a different coder) and examine them for overall consistency before submitting them to the data management team.
2. The data management team passes the received spreadsheets through an R script that checks the consistency of the identifiers of the regional government announcing the policy and the regions to which the policies apply, the level of government and the announcing region, the report and effect dates, and detect other potential inconsistencies in the data. All inconsistencies are fixed in the coding spreadsheets or referred back to the GR curators.
3. GR curators address the queries received from data management team and return country files to data management.
4. Upon combining the data into the operational storage, the data management team generates spreadsheets with the color-coded trajectory of the policy stringency in specific categories and sends them to the GR curators, who further analytically examine the consistency of the stringency and, together with the data management team, resolve the inconsistencies.
5. Data management team pools country files into a cross-national dataset.

### Validation of the index construction

**Fig. 2:**
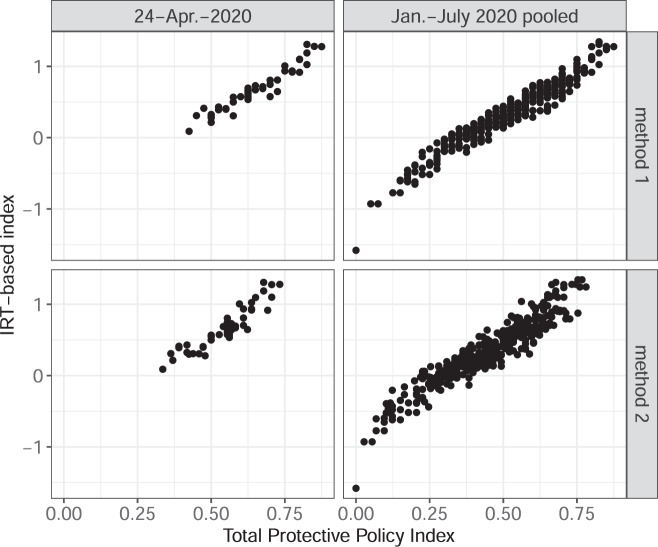
PPI and IRT-based index in the US states. This figure presents scatterplots with the state-level Total Protective Policy Index (on the horizontal axis) and an index that relies on the same components but is generated through a Bayesian IRT model (vertical axis). The scatterplots are organized in four panels: 2 panels for each method of PPI calculations and for each subsample of the dataset. Each marker represents a US state-day.

**Fig. 3:**
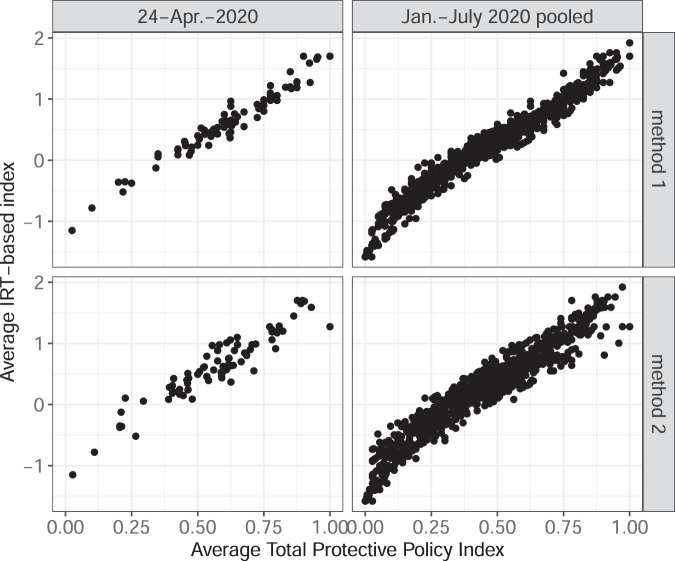
Country-level average PPI and IRT-based index. This figure presents scatterplots with the Average Total Protective Policy Index (on the horizontal axis) and an index that relies on the same components but is generated through a Bayesian IRT model (vertical axis). The scatterplots are organized in four panels: 2 panels for each method of PPI calculations and for each subsample of the dataset. Each marker represents a country-day.

We conduct three types of validation of the construction of the PPI. First, we used an alternative procedure for combing information from the policy measures and compared it against our index. We converted region-specific values of the combined stringency of policy measures within categories included in method 1 (with the addition of the mandate for social distancing measures) into three-level ordinal scales and estimated an ordinal IRT model^16^. Such an approach implicitly views the observed policy states as the consequences of the overall policy stringency. While driven by different understandings of the connection between the observed policies and the overall policy stringency, the IRT-based index and the PPI are closely correlated. Figure 2 shows the relationship between PPI values and the values of the IRT-based index in the US states. It reveals a positive correlation between them both in a pooled sample encompassing data between January and July 2020 and in the snapshot sample as of April 24, 2020. Indeed, method 1 index is correlated with the IRT-based index at 0.983, and method 2 index is correlated with it at 0.971. Figure 3 displays a similar relationship between the country-level aggregates. The correlation between methods 1 and 2 PPI and the IRT-based index is at 0.978 and 0.969 respectively.

**Fig. 4:**
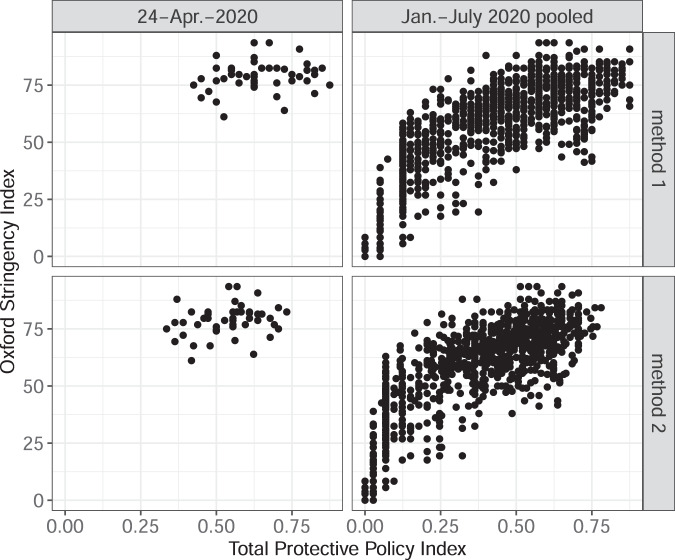
PPI and Oxford Stringency Index in the US states. This figure presents scatterplots with the state-level Total Protective Policy Index (on the horizontal axis) and the value of the Oxford Stringency Index (vertical axis). The scatterplots are organized in four panels: 2 panels for each method of PPI calculations and for each subsample of the dataset. Each marker represents a US state-day.

**Fig. 5:**
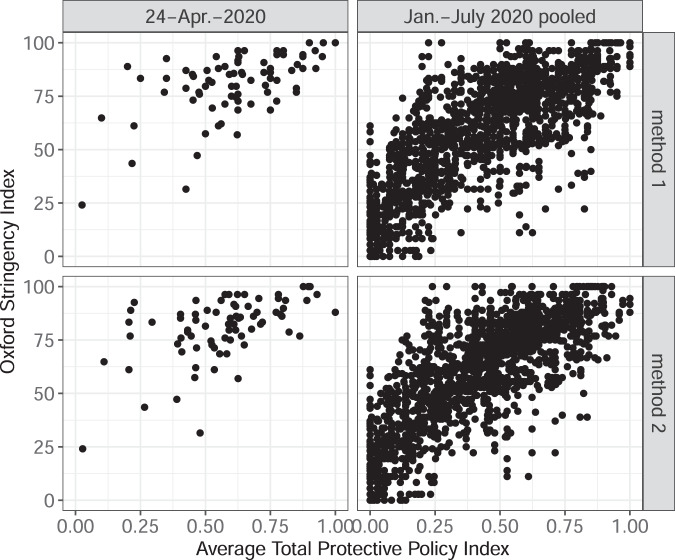
Country-level average PPI and Oxford Stringency Index. This figure presents scatterplots with the Average Total Protective Policy Index (on the horizontal axis) and the value of the Oxford Stringency Index (vertical axis). The scatterplots are organized in four panels: 2 panels for each method of PPI calculations and for each subsample of the dataset. Each marker represents a country-day.

Second, we compare the values of the PPI against the Stringency Index produced by the Oxford COVID-19 Government Response Tracker^17^, one of the alternative measures of policy stringency. Figure 4 shows the relationship between PPI and the Oxford Stringency Index in the US states. The correlation is positive both in the pooled sample with all overlapping observations (it is at 0.889 and 0.882 for methods 1 and 2 respectively) and a sample (as of April 24, 2020; 0.213 and 0.240 for methods 1 and 2 respectively), but is significantly weaker in the April 24, 2020 snapshot. The Oxford Stringency Index appears to have less variation than PPI on that date. Figure 5 shows the relationship between PPI and the Oxford Stringency Index in a country-level sample. The correlation is positive (it is at 0.855 and 0.851 in the pooled sample for methods 1 and 2 respectively; it is at 0.569 and 0.541 as of April 24, 2020).

It is a healthy norm in social sciences to have competing data sets and for researchers to triangulate and compare. Multiple independently developed data-collection projects targeting the same or similar phenomena enrich the research community by offering opportunities to average out project-specific biases. While a project that relies on one dataset inevitably inherits the premises and biases of the authors of that dataset, projects that peruse multiple datasets can triangulate their findings for more valid conclusions. The fact that the stringency indices generated by our team and that of the OxGRT are correlated adds to the validity of both. The unique disciplinary value to our project comes from the purposeful collection of the institutional origins of the COVID-19 policies. Finally, our stringency index was designed to reflect the beliefs about the effectiveness of the public health measures in inhibiting the spread of the coronavirus. That is why we also relate its values to the objective rate of the spread of the infection. As described by Shvetsova *et al*., PPI was negatively related to the rate of the spread of the virus in the US in 2020^8^. According to their estimates, a 10 percentage point increase in the method 1 PPI was associated with an about 8 percent reduction in the rate of the virus spread. Although these are too general to assess item-by-item efficacy, and while the American case cannot be seen as a reflection of a global pattern, this suggests a possibility for future transdisciplinary use of this data.

## Supplementary information

Appendix

## Data Availability

The code used to produce our calculations is available at https://github.com/COVID-policy-response-lab/PPI-data.
