# Supplementary material for: Protective Policy Index (PPI) global dataset of origins and stringency of COVID 19 mitigation policies
Source: Sci Data. 2022 Jun 16;9:319. doi: 10.1038/s41597-022-01437-9 (PMC9203541; doi:10.1038/s41597-022-01437-9)
Supplement: Supplementary file 1 — Appendix [file 41597_2022_1437_MOESM1_ESM.docx]

**Appendix to: Protective Policy Index (PPI), a Global dataset of origins and stringency of COVID 19 mitigation policies**

**Authors**

Olga Shvetsova^1^, Andrei Zhirnov^2^, Abdul Basit Adeel^3^, Mert Can Bayar^1^, Onsel Gurel Bayrali^1^, Michael Catalano^1^, Olivia Catalano^4^, Hyoungrohk Chu^1^, Frank Giannelli^5^, Ezgi Muftuoglu^1^, Dina Rosenberg^6^, Didem Seyis^1^, Bradley Skopyk^1^, Julie VanDusky-Allen^7^, Tianyi Zhao^1^

**Affiliations**

1. Binghamton University, Binghamton, New York, USA
2. University of Exeter, Exeter, Devon, UK
3. Penn State University, State College, Pennsylvania, USA
4. MS-MPH, unaffiliated researcher, USA
5. Rutgers, the State University of New Jersey, Piscataway, New Jersey, USA
6. National Research University-Higher School of Economics, Moscow, Russia
7. Boise State University, Boise, Idaho, USA

Corresponding author: Olga Shvetsova shvetso@binghamton.edu

A1**: Published research with previous versions of the dataset**

Earlier versions of this dataset were used in the analyses presented in the following published and unpublished research articles. Shvetsova et al. find that democracies were more active as the autocracies and decentralized polities were at least as active as the centralized polities in their responses to the COVID-19 pandemic in spring 2020.^1^ Adeel et al. observe that in the US and Canada the subnational government take the lead in the COVID-19 response^2^. Shvetsova et al. explore the variation in the national/subnational contribution across the federations to find the influence of the constitutional role of each level of government in protecting public health^3^.

VanDusky-Allen, Utych, and Catalano (2021) find that during the initial re-opening period, US state COVID-19 policies influenced how Democrats evaluated their state's response to the pandemic^4^. Shvetsova et al. produce an application of PPI to analyzethe dynamics of COVID-19 cases^5^. Treating PPI as a complex measure of the public health regime (as opposed to the binary indicators of specific policies), they find that a 10 percentage point increase in PPI was associated with an about 8 percent drop in the rate of coronavirus spread in the US states in 2020.

References:

1. Shvetsova O, Zhirnov A, VanDusky-Allen J, et al. Institutional origins of protective COVID-19 public health policy responses: informational and authority redundancies and policy stringency. *J Political Institutions Political Economy*. 2020; 4(1): 585-613. <https://dx.doi.org/10.1561/113.00000023>.
2. Adeel AB, Catalano M, Catalano O., et al. COVID-19 Policy response and the rise of the sub-national governments. *Can Public Policy*. 2020; 46(4): 565-584. <https://doi.org/10.3138/cpp.2020-101>.
3. Shvetsova O, VanDusky-Allen J, Zhirnov A, Adeel AB, Catalano M, Catalano O, Giannelli F, Muftuoglu E, Rosenberg D, Sezgin MH, Zhao T. Federal Institutions and Strategic Policy Responses to COVID-19 Pandemic. Frontiers in Political Science. 2021a Jun 22;3:66. <https://doi.org/10.3389/fpos.2021.631363>.
4. VanDusky-Allen JA, Utych SM, Catalano M. Partisanship, Policy, and Americans’ Evaluations of State-Level COVID-19 Policies Prior to the 2020 Election. Political Research Quarterly. 2021:10659129211056374.
5. Shvetsova O, Zhirnov A, Giannelli FR, Catalano MA, Catalano O. Governor's Party, Policies, and COVID-19 Outcomes: Further Evidence of an Effect. American journal of preventive medicine. 2021b Oct 11. <https://doi.org/10.1016/j.amepre.2021.09.003>.

A2:

**Substantive criteria in the coding of NMI policies**

**Method 1: Coding criteria and stringency levels of component policies**

| Dimension | Description | Value |  | Strength level | Notes |
| --- | --- | --- | --- | --- | --- |
| borders.air_bord | Border closures: closure of air borders | 1.00 | 3 | All air borders are closed | Both international and domestic air borders are closed for non-citizen people travel; does not apply to trade |
|  |  | 0.67 | 2 | All international air borders are closed | No international non-citizen people air travel is allowed, regardless of whether origin countries are receiving; does not apply to trade |
|  |  | 0.33 | 1 | Air borders are closed for select countries | No international non-citizen people air travel is allowed with select countries, regardless of whether origin countries are receiving; does not apply to trade |
|  |  | 0.00 | 0 | All air borders are open | No air travel restrictions, but can require testing or quarantines |
| borders.land_bord | Border closures: closure of land borders | 1.00 | 3 | All land borders are closed | Both international and domestic land borders are closed for non-citizen people travel; does not apply to trade |
|  |  | 0.67 | 2 | All international land borders are closed | No international non-citizen people land travel is allowed, regardless of whether origin countries are receiving; does not apply to trade |
|  |  | 0.33 | 1 | Land borders with select countries are closed | No international non-citizen people land travel is allowed with select countries, regardless of whether origin countries are receiving; does not apply to trade |
|  |  | 0.00 | 0 | All land borders are open | No land travel restrictions, but can require testing or quarantines |
| borders.sea_bord | Border closures: closure of sea borders | 1.00 | 3 | All sea borders are closed | Both international and domestic sea borders are closed for non-citizen people travel; does not apply to trade |
|  |  | 0.67 | 2 | All international sea borders are closed | No international non-citizen people sea travel is allowed, regardless of whether origin countries are receiving; does not apply to trade |
|  |  | 0.33 | 1 | Sea borders with select countries are closed | No international non-citizen people sea travel is allowed with select countries, regardless of whether origin countries are receiving; does not apply to trade |
|  |  | 0.00 | 0 | All sea borders are open | No sea travel restrictions, but can require testing or quarantines |
| emerg.all | State of emergency | 1.00 | 1 | State of emergency | Announced; language can vary; curator will re-check |
|  |  | 0.00 | 0 | No State of emergency | Not announced |
| ind_locat.ind_mob | Individual location: restricted individual mobility | 1.00 | 5 | Lockdown (permission required | Lockdown (permission/ app to exit home is required); note that sometimes may be labeled “curfew” in policy documents |
|  |  | 0.80 | 4 | Stay-at-home for all (no permission required | Stay-at-home for all (no permission required; and unless they are in permitted groups) |
|  |  | 0.40 | 2 | Stay-at-home order just for specified groups | Stay-at-home order just for specified groups |
|  |  | 0.20 | 1 | No stay at home restrictions, curfew | Word “curfew” for times when people are not allowed to be outside (unless they are in permitted groups); check that they are not calling curfew what is in fact a lockdown |
|  |  | 0.00 | 0 | No stay at home or curfew restrictions | No restrictions |
| ind_locat.med_stay | Individual location: conditional self-isolation | 1.00 | 3 | Mandatory quarantine if diagnosed, exposed, or travelled | Hotel placement +monitoring; tracking through apps at reported address; placement in hospitals; somebody came to their house; punished for violating if discovered |
|  |  | 0.33 | 1 | Self-isolation mandated for exposure and travel | You are told to stay home but no enforcement mechanism is specified |
|  |  | 0.00 | 0 | No mandatory quarantines or self-isolation | Above mechanisms are not used |
| ind_locat.publ_tr | Individual location: closure of public transportation | 1.00 | 1 | Public transportation closed (except emergency routes) | Note: possible difficulty in identifying when routes start reopening; may be reported only locally to passengers of specific routes  Also includes when categories of patients are banned (e.g., with COVID themselves or in family, via apps) |
|  |  | 0.00 | 0 | Public transportation not closed | Public transportation is fully open, but may require masks |
| places.gov_offs | Closures of places of human congregation: closure of government offices | 1.00 | 1 | Government offices are closed | Includes when some are still open |
|  |  | 0.00 | 0 | Government offices are open | Includes when most (exceptions are those serving vulnerable groups) are open |
| places.ne_busn | Closures of places of human congregation: closure of non-essential businesses | 1.00 | 1 | Nonessential businesses are closed | Usually there is a list of businesses that are deemed essential; can vary by country and state/region |
|  |  | 0.00 | 0 | Nonessential businesses are open | Includes when most (exceptions are those serving vulnerable groups) are open |
| places.restrts | Closures of places of human congregation: closure of restaurants | 1.00 | 1 | Restaurants are closed | Restaurants are fully closed (except delivery and take-out) |
|  |  | 0.00 | 0 | Restaurants are open | No restrictions |
| places.venues | Closures of places of human congregation: closure of venues of entertainment and leisure | 1.00 | 1 | Entertainment venues /stadiums are closed | All entertainment venues /stadiums are closed |
|  |  | 0.00 | 0 | Entertainment venues /stadiums are open | Also includes when select outdoor venues are open earlier than the rest |
| places.wfh | Closures of places of human congregation: working from home requirement | 1.00 | 1 | Working from home is required | For all non-essential workers or for a specified percentage |
|  |  | 0.00 | 0 | No work-from-home requirement |  |
| soc_and_schls.schools | Closure of schools and restrictions on social gatherings: closure of schools | 1.00 | 2 | Full closure of K12 schools |  |
|  |  | 0.50 | 1 | Partial closure of K12 schools | Only some grades/ages attend in-person; masks and social distancing may be required |
|  |  | 0.00 | 0 | K12 schools are not required to closed | Masks and social distancing may be required |
| soc_and_schls.soc_gath | Closure of schools and restrictions on social gatherings: limits on size of social gatherings | 1.00 | 4 | All social gatherings are prohibited | Includes when <10 are permitted  If different restrictions depending on where, choose the easiest restriction.  Includes when only household can meet. |
|  |  | 0.75 | 3 | Gatherings of 10 and more people are prohibited | Includes when <50 are permitted  If different restrictions depending on where, choose the easiest restriction. |
|  |  | 0.50 | 2 | Gatherings of 50 and more people are prohibited | Includes when <100 are permitted  If different restrictions depending on where, choose the easiest restriction. |
|  |  | 0.25 | 1 | Gatherings of 100 and more people are prohibited | If different restrictions depending on where, choose the easiest restriction.  Also if restriction policy announced with no number |
|  |  | 0.00 | 0 | Social gatherings are not restricted |  |
| masks.all | Mandatory wearing of personal protective equipment | 1.00 | 1 | Mandatory wearing of masks/PPE | Mandatory wearing of masks/PPE either everywhere except at home or in indoor public spaces or by enployees |
|  |  | 0.00 | 0 | No PPE/masks mandate |  |

**Method 2 stringency levels of component policies**

| Dimension | Description | Value |  | Criterion | Notes |
| --- | --- | --- | --- | --- | --- |
| borders.all | Border closures | 1.00 | 8 | All borders are closed | Both international and domestic borders are closed for non-citizen people travel |
|  |  | 0.63 | 5 | All international borders are closed | No international non-citizen people travel is allowed, regardless of whether origin countries are receiving, does not apply to trade |
|  |  | 0.25 | 2 | Borders are closed for select countries | No international non-citizen people travel is allowed with select countries, regardless of whether origin countries are receiving, does not apply to trade |
|  |  | 0.00 | 0 | All borders are open | No travel restrictions, but can require testing or quarantines |
| emerg.all | State of emergency | 1.00 | 1 | State of emergency | Announced; language can vary; curator will re-check |
|  |  | 0.00 | 0 | No State of emergency | Not announced |
| ind_locat.ind_mob | Individual location: restricted individual mobility | 1.00 | 10 | Lockdown (permission to exit home is required) | Lockdown (permission/ app to exit home is required); note that sometimes may be labeled “curfew” in policy documents |
|  |  | 0.80 | 8 | Stay-at-home for all (no permission required) | Stay-at-home for all (no permission required; and unless they are in permitted groups) |
|  |  | 0.40 | 4 | Stay-at-home order just for specified groups | Stay-at-home order just for specified groups |
|  |  | 0.10 | 1 | No stay at home restrictions, curfew | Word “curfew” for times when people are not allowed to be outside (unless they are in permitted groups); check that they are not calling curfew what is in fact a lockdown |
|  |  | 0.00 | 0 | No stay at home or curfew restrictions | No restrictions |
| ind_locat.med_quar | Individual location: mandatory quarantine | 1.00 | 4 | Mandatory quarantine if diagnosis/exposure/travel | Hotel placement +monitoring; tracking through apps at reported address; placement in hospitals; somebody came to their house; punished for violating if discovered |
|  |  | 0.50 | 2 | Mandatory quarantine if diagnosis /exposure only | Hotel placement +monitoring; tracking through apps at reported address; placement in hospitals; somebody came to their house; punished for violating if discovered |
|  |  | 0.25 | 1 | Mandatory quarantine for diagnosis only | Hotel placement +monitoring; tracking through apps at reported address; placement in hospitals; somebody came to their house; punished for violating if discovered |
|  |  | 0.00 | 0 | Mandatory quarantines not imposed | Above mechanisms are not used |
| ind_locat.med_stay | Individual location: conditional self-isolation | 1.00 | 1 | Self-isolation is mandated after exposure and travel | You are told to stay home but no enforcement mechanism is specified |
|  |  | 0.00 | 0 | Self-isolation not mandated | No mention of the above |
| ind_locat.publ_tr | Individual location: closure of public transportation | 1.00 | 2 | Public transportation closed (except emergency routes) | Note: possible difficulty in identifying when routes start reopening; may be reported only locally to passengers of specific routes |
|  |  | 0.50 | 1 | Public transportation limited to share of capacity/time/users | Also includes when categories of patients are banned (e.g., with COVID themselves or in family, via apps) |
|  |  | 0.00 | 0 | Public transportation is fully open |  |
| places.gov_offs | Closures of places of human congregation: closure of government offices | 1.00 | 1 | Government offices are closed | Includes when some are still open |
|  |  | 0.00 | 0 | Government offices are open | Includes when most are open |
| places.ne_busn | Closures of places of human congregation: closure of non-essential businesses | 1.00 | 4 | Nonessential businesses are closed | Essential category may be broader or narrower, depending on country or subnational unit |
|  |  | 0.25 | 1 | Nonessential businesses are closed (except curbside service/ outdoor construction) | Essential category may be broader or narrower, depending on country or subnational unit |
|  |  | 0.00 | 0 | Nonessential businesses are open | Also includes when select non-essential are open earlier than the rest |
| places.restrts | Closures of places of human congregation: closure of restaurants | 1.00 | 4 | Restaurants are fully closed (except delivery and take-out) |  |
|  |  | 0.75 | 3 | Restaurants are opened for outdoor, not indoor |  |
|  |  | 0.25 | 1 | Restaurants are opened, reduced indoor capacity | Also may include when categories of patients are banned (e.g., with COVID themselves or in family, via apps) |
|  |  | 0.00 | 0 | Restaurants are fully open | Also may include when categories of patients are banned (e.g., with COVID themselves or in family, via apps) |
| places.venues | Closures of places of human congregation: closure of venues of entertainment and leisure | 1.00 | 2 | Entertainment venues /stadiums are closed |  |
|  |  | 0.50 | 1 | Only outdoors entertainment venues are open | Also includes when select outdoor venues are open earlier than the rest |
|  |  | 0.00 | 0 | Entertainment venues /stadiums are open | Also includes when select indoor venues are open earlier than the rest |
| places.wfh | Closures of places of human congregation: working from home requirement | 1.00 | 1 | Working from home is required | For all non-essential workers or for a specified percentage |
|  |  | 0.00 | 0 | No work-from-home requirement | No work-from-home requirement |
| soc_and_schls.schools | Closure of schools and restrictions on social gatherings: closure of schools | 1.00 | 2 | Full closure of K12 schools | Full closure of K12 schools |
|  |  | 0.50 | 1 | Partial closure of K12 schools | Only some grades/ages attend in-person |
|  |  | 0.00 | 0 | K12 schools are not required to closed | K12 schools are not required to closed |
| soc_and_schls.soc_gath | Closure of schools and restrictions on social gatherings: limits on size of social gatherings | 1.00 | 4 | All social gatherings are prohibited | Includes when <10 are permitted  If different restrictions depending on where, choose the easiest restriction.  Includes when only household can meet. |
|  |  | 0.75 | 3 | Gatherings of 10 and more people are prohibited | Includes when <50 are permitted  If different restrictions depending on where, choose the easiest restriction. |
|  |  | 0.50 | 2 | Gatherings of 50 and more people are prohibited | Includes when <100 are permitted  If different restrictions depending on where, choose the easiest restriction. |
|  |  | 0.25 | 1 | Gatherings of 100 and more people are prohibited | If different restrictions depending on where, choose the easiest restriction.  Also if restriction policy announced with no number |
|  |  | 0.00 | 0 | Social gatherings are not restricted | Social gatherings are not restricted |
| med_mandate.dist_mand | Medical mandate: social distancing mandates | 1.00 | 2 | Safe distance 1.5-2m outdoors and in public spaces/transport | Safe distance 1.5-2m outdoors and in public spaces/transport |
|  |  | 0.50 | 1 | Safe distance 1.5-2m in business/organizations | Safe distance 1.5-2m in business/organizations |
|  |  | 0.00 | 0 | No safe distance mandate | No safe distance mandate |
| med_mandate.masks | Medical mandate: mandatory wearing of personal protective equipment | 1.00 | 8 | Mandatory wearing of masks/PPE everywhere except at home | Mandatory wearing of masks/PPE everywhere except at home |
|  |  | 0.63 | 5 | Mandatory wearing of masks/PPE indoors public spaces | Mandatory wearing of masks/PPE indoors public spaces |
|  |  | 0.25 | 2 | Mandatory wearing of masks/PPE required for employees | Mandatory wearing of masks/PPE required for employees |
|  |  | 0 | 0 | No PPE/masks mandate | No PPE/masks mandate |

A3: **Public Health Protective Policy Index (PPI) Dataset: Full Codebook**

**MAIN DATASETS**

**PPI_country_m1:** stores information about active national and subnational policies in each country on each day of observation, with the policy variables constructed according to the first method below and aggregated over the subnational units.

**PPI_regions_ZZ_m1:** stores information about active national and subnational policies in each region of country ZZ on each day of observation, with the policy variables constructed according to the first method below.

**PPI_country_m2:** stores information about active national and subnational policies in each country on each day of observation, with the policy variables constructed according to the second method below and aggregated over the subnational units.

**PPI_regions_ZZ_m2:** stores information about active national and subnational policies in each region of country ZZ on each day of observation, with the policy variables constructed according to the second method below.

**Country-level Protective Policy Index Method 1 (PPI-M1) dataset**

|  | Variable name | Description | Construction | Range |
| --- | --- | --- | --- | --- |
| **Case Identification variables:** | | | | |
|  | cname | Country name |  |  |
|  | isocode | ISO 3166 country code |  |  |
|  | isoabbr | country abbreviation according to ISO 3166 |  |  |
|  | date | date |  |  |
| **Protective Policy Index Method 1 (PPI-M1) calculated variables:** | | | | |
|  | ppi.all.tot.ave | Protective Policy Index (Both levels) | Population-weighted average of region-specific values of ppi.all.tot | [0,1] |
|  | ppi.all.nat.ave | Protective Policy Index (National) | Population-weighted average of region-specific values of ppi.all.nat | [0,1] |
|  | ppi.all.reg.ave | Protective Policy Index (Regional) | Population-weighted average of region-specific values of ppi.all.reg | [0,1] |
| **Borders:** | | | | |
|  | borders.all.tot.ave | Border closures: all components (Both levels) | Population-weighted average of region-specific values of borders.all.tot | [0,1] |
|  | borders.all.nat.ave | Border closures: all components (National) | Population-weighted average of region-specific values of borders.all.nat | [0,1] |
|  | borders.all.reg.ave | Border closures: all components (Regional) | Population-weighted average of region-specific values of borders.all.reg | [0,1] |
|  | borders.air_bord.tot.ave | Border closures: closure of air borders (Both levels) | Population-weighted average of region-specific values of borders.air_bord.tot | [0,1] |
|  | borders.air_bord.nat.ave | Border closures: closure of air borders (National) | Population-weighted average of region-specific values of borders.air_bord.nat | [0,1] |
|  | borders.air_bord.reg.ave | Border closures: closure of air borders (Regional) | Population-weighted average of region-specific values of borders.air_bord.reg | [0,1] |
|  | borders.land_bord.tot.ave | Border closures: closure of land borders (Both levels) | Population-weighted average of region-specific values of borders.land_bord.tot | [0,1] |
|  | borders.land_bord.nat.ave | Border closures: closure of land borders (National) | Population-weighted average of region-specific values of borders.land_bord.nat | [0,1] |
|  | borders.land_bord.reg.ave | Border closures: closure of land borders (Regional) | Population-weighted average of region-specific values of borders.land_bord.reg | [0,1] |
|  | borders.sea_bord.tot.ave | Border closures: closure of sea borders (Both levels) | Population-weighted average of region-specific values of borders.sea_bord.tot | [0,1] |
|  | borders.sea_bord.nat.ave | Border closures: closure of sea borders (National) | Population-weighted average of region-specific values of borders.sea_bord.nat | [0,1] |
|  | borders.sea_bord.reg.ave | Border closures: closure of sea borders (Regional) | Population-weighted average of region-specific values of borders.sea_bord.reg | [0,1] |
| **State of emergency:** | | | | |
|  | emerg.all.tot.ave | State of emergency (Both levels) | Population-weighted average of region-specific values of emerg.all.tot | [0,1] |
|  | emerg.all.nat.ave | State of emergency (National) | Population-weighted average of region-specific values of emerg.all.nat | [0,1] |
|  | emerg.all.reg.ave | State of emergency (Regional) | Population-weighted average of region-specific values of emerg.all.reg | [0,1] |
| **Individual location restrictions:** | | | | |
|  | ind_locat.all.tot.ave | Individual location: all components (Both levels) | Population-weighted average of region-specific values of ind_locat.all.tot | [0,1] |
|  | ind_locat.all.nat.ave | Individual location: all components (National) | Population-weighted average of region-specific values of ind_locat.all.nat | [0,1] |
|  | ind_locat.all.reg.ave | Individual location: all components (Regional) | Population-weighted average of region-specific values of ind_locat.all.reg | [0,1] |
|  | ind_locat.ind_mob.tot.ave | Individual location: restricted individual mobility (Both levels) | Population-weighted average of region-specific values of ind_locat.ind_mob.tot | [0,1] |
|  | ind_locat.ind_mob.nat.ave | Individual location: restricted individual mobility (National) | Population-weighted average of region-specific values of ind_locat.ind_mob.nat | [0,1] |
|  | ind_locat.ind_mob.reg.ave | Individual location: restricted individual mobility (Regional) | Population-weighted average of region-specific values of ind_locat.ind_mob.reg | [0,1] |
|  | ind_locat.med_stay.tot.ave | Individual location: conditional self-isolation (Both levels) | Population-weighted average of region-specific values of ind_locat.med_stay.tot | [0,1] |
|  | ind_locat.med_stay.nat.ave | Individual location: conditional self-isolation (National) | Population-weighted average of region-specific values of ind_locat.med_stay.nat | [0,1] |
|  | ind_locat.med_stay.reg.ave | Individual location: conditional self-isolation (Regional) | Population-weighted average of region-specific values of ind_locat.med_stay.reg | [0,1] |
|  | ind_locat.publ_tr.tot.ave | Individual location: closure of public transportation (Both levels) | Population-weighted average of region-specific values of ind_locat.publ_tr.tot | [0,1] |
|  | ind_locat.publ_tr.nat.ave | Individual location: closure of public transportation (National) | Population-weighted average of region-specific values of ind_locat.publ_tr.nat | [0,1] |
|  | ind_locat.publ_tr.reg.ave | Individual location: closure of public transportation (Regional) | Population-weighted average of region-specific values of ind_locat.publ_tr.reg | [0,1] |
| **Closure of places of congregation:** | | | | |
|  | places.all.tot.ave | Closures of places of human congregation: all components (Both levels) | Population-weighted average of region-specific values of places.all.tot | [0,1] |
|  | places.all.nat.ave | Closures of places of human congregation: all components (National) | Population-weighted average of region-specific values of places.all.nat | [0,1] |
|  | places.all.reg.ave | Closures of places of human congregation: all components (Regional) | Population-weighted average of region-specific values of places.all.reg | [0,1] |
|  | places.gov_offs.tot.ave | Closures of places of human congregation: closure of government offices (Both levels) | Population-weighted average of region-specific values of places.gov_offs.tot | [0,1] |
|  | places.gov_offs.nat.ave | Closures of places of human congregation: closure of government offices (National) | Population-weighted average of region-specific values of places.gov_offs.nat | [0,1] |
|  | places.gov_offs.reg.ave | Closures of places of human congregation: closure of government offices (Regional) | Population-weighted average of region-specific values of places.gov_offs.reg | [0,1] |
|  | places.ne_busn.tot.ave | Closures of places of human congregation: closure of non-essential businesses (Both levels) | Population-weighted average of region-specific values of places.ne_busn.tot | [0,1] |
|  | places.ne_busn.nat.ave | Closures of places of human congregation: closure of non-essential businesses (National) | Population-weighted average of region-specific values of places.ne_busn.nat | [0,1] |
|  | places.ne_busn.reg.ave | Closures of places of human congregation: closure of non-essential businesses (Regional) | Population-weighted average of region-specific values of places.ne_busn.reg | [0,1] |
|  | places.restrts.tot.ave | Closures of places of human congregation: closure of restaurants (Both levels) | Population-weighted average of region-specific values of places.restrts.tot | [0,1] |
|  | places.restrts.nat.ave | Closures of places of human congregation: closure of restaurants (National) | Population-weighted average of region-specific values of places.restrts.nat | [0,1] |
|  | places.restrts.reg.ave | Closures of places of human congregation: closure of restaurants (Regional) | Population-weighted average of region-specific values of places.restrts.reg | [0,1] |
|  | places.venues.tot.ave | Closures of places of human congregation: closure of venues of entertainment and leisure (Both levels) | Population-weighted average of region-specific values of places.venues.tot | [0,1] |
|  | places.venues.nat.ave | Closures of places of human congregation: closure of venues of entertainment and leisure (National) | Population-weighted average of region-specific values of places.venues.nat | [0,1] |
|  | places.venues.reg.ave | Closures of places of human congregation: closure of venues of entertainment and leisure (Regional) | Population-weighted average of region-specific values of places.venues.reg | [0,1] |
|  | places.wfh.tot.ave | Closures of places of human congregation: working from home requirement (Both levels) | Population-weighted average of region-specific values of places.wfh.tot | [0,1] |
|  | places.wfh.nat.ave | Closures of places of human congregation: working from home requirement (National) | Population-weighted average of region-specific values of places.wfh.nat | [0,1] |
|  | places.wfh.reg.ave | Closures of places of human congregation: working from home requirement (Regional) | Population-weighted average of region-specific values of places.wfh.reg | [0,1] |
| **Schools and social gatherings:** | | | | |
|  | soc_and_schls.all.tot.ave | Closure of schools and restrictions on social gatherings: all components (Both levels) | Population-weighted average of region-specific values of soc_and_schls.all.tot | [0,1] |
|  | soc_and_schls.all.nat.ave | Closure of schools and restrictions on social gatherings: all components (National) | Population-weighted average of region-specific values of soc_and_schls.all.nat | [0,1] |
|  | soc_and_schls.all.reg.ave | Closure of schools and restrictions on social gatherings: all components (Regional) | Population-weighted average of region-specific values of soc_and_schls.all.reg | [0,1] |
|  | soc_and_schls.schools.tot.ave | Closure of schools and restrictions on social gatherings: closure of schools (Both levels) | Population-weighted average of region-specific values of soc_and_schls.schools.tot | [0,1] |
|  | soc_and_schls.schools.nat.ave | Closure of schools and restrictions on social gatherings: closure of schools (National) | Population-weighted average of region-specific values of soc_and_schls.schools.nat | [0,1] |
|  | soc_and_schls.schools.reg.ave | Closure of schools and restrictions on social gatherings: closure of schools (Regional) | Population-weighted average of region-specific values of soc_and_schls.schools.reg | [0,1] |
|  | soc_and_schls.soc_gath.tot.ave | Closure of schools and restrictions on social gatherings: limits on size of social gatherings (Both levels) | Population-weighted average of region-specific values of soc_and_schls.soc_gath.tot | [0,1] |
|  | soc_and_schls.soc_gath.nat.ave | Closure of schools and restrictions on social gatherings: limits on size of social gatherings (National) | Population-weighted average of region-specific values of soc_and_schls.soc_gath.nat | [0,1] |
|  | soc_and_schls.soc_gath.reg.ave | Closure of schools and restrictions on social gatherings: limits on size of social gatherings (Regional) | Population-weighted average of region-specific values of soc_and_schls.soc_gath.reg | [0,1] |
| **Personal protective equipment:** | | | | |
|  | masks.all.tot.ave | Mandatory wearing of personal protective equipment (Both levels) | Population-weighted average of region-specific values of masks.all.tot | [0,1] |
|  | masks.all.nat.ave | Mandatory wearing of personal protective equipment (National) | Population-weighted average of region-specific values of masks.all.nat | [0,1] |
|  | masks.all.reg.ave | Mandatory wearing of personal protective equipment (Regional) | Population-weighted average of region-specific values of masks.all.reg | [0,1] |

**Sub-national level Protective Policy Indices by Method 1 (PPI-M1)**

|  | Variable name | Description | Construction | Range |
| --- | --- | --- | --- | --- |
| **Case Identification variables:** | | | | |
|  | cname | Country name |  |  |
|  | isocode | ISO 3166 country code |  |  |
|  | isoabbr | country abbreviation according to ISO 3166 |  |  |
|  | state_province | the name of the administrative division where the policy applies |  |  |
|  | iso_state | ISO 3166 code of the administrative division where the policy applies |  |  |
|  | date | date |  |  |
| **Protective Policy Index Method 1 (PPI-M1) calculated variables:** | | | | |
|  | ppi.all.tot | Protective Policy Index (Both levels) | (9*borders.all.tot+3*emerg.all.tot+10*ind_locat.all.tot+8*places.all.tot+8*soc_and_schls.all.tot+2*masks.all.tot)/40 | [0,1] |
|  | ppi.all.nat | Protective Policy Index (National) | (9*borders.all.nat+3*emerg.all.nat+10*ind_locat.all.nat+8*places.all.nat+8*soc_and_schls.all.nat+2*masks.all.nat)/40 | [0,1] |
|  | ppi.all.reg | Protective Policy Index (Regional) | (9*borders.all.reg+3*emerg.all.reg+10*ind_locat.all.reg+8*places.all.reg+8*soc_and_schls.all.reg+2*masks.all.reg)/40 | [0,1] |
| **Borders:** | | | | |
|  | borders.all.tot | Border closures: all components (Both levels) | (3*borders.air_bord.tot+3*borders.land_bord.tot+3*borders.sea_bord.tot)/9 | [0,1] |
|  | borders.all.nat | Border closures: all components (National) | (3*borders.air_bord.nat+3*borders.land_bord.nat+3*borders.sea_bord.nat)/9 | [0,1] |
|  | borders.all.reg | Border closures: all components (Regional) | (3*borders.air_bord.reg+3*borders.land_bord.reg+3*borders.sea_bord.reg)/9 | [0,1] |
|  | borders.air_bord.tot | Border closures: closure of air borders (Both levels) | max(borders.air_bord.nat,borders.air_bord.reg) | [0,1] |
|  | borders.air_bord.nat | Border closures: closure of air borders (National) | directly coded | [0,1] |
|  | borders.air_bord.reg | Border closures: closure of air borders (Regional) | directly coded | [0,1] |
|  | borders.land_bord.tot | Border closures: closure of land borders (Both levels) | max(borders.land_bord.nat,borders.land_bord.reg) | [0,1] |
|  | borders.land_bord.nat | Border closures: closure of land borders (National) | directly coded | [0,1] |
|  | borders.land_bord.reg | Border closures: closure of land borders (Regional) | directly coded | [0,1] |
|  | borders.sea_bord.tot | Border closures: closure of sea borders (Both levels) | max(borders.sea_bord.nat,borders.sea_bord.reg) | [0,1] |
|  | borders.sea_bord.nat | Border closures: closure of sea borders (National) | directly coded | [0,1] |
|  | borders.sea_bord.reg | Border closures: closure of sea borders (Regional) | directly coded | [0,1] |
| **State of emergency:** | | | | |
|  | emerg.all.tot | State of emergency (Both levels) | max(emerg.all.nat,emerg.all.reg) | [0,1] |
|  | emerg.all.nat | State of emergency (National) | directly coded | [0,1] |
|  | emerg.all.reg | State of emergency (Regional) | directly coded | [0,1] |
| **Individual location restrictions:** | | | | |
|  | ind_locat.all.tot | Individual location: all components (Both levels) | (5*ind_locat.ind_mob.tot+3*ind_locat.med_stay.tot+2*ind_locat.publ_tr.tot)/10 | [0,1] |
|  | ind_locat.all.nat | Individual location: all components (National) | (5*ind_locat.ind_mob.nat+3*ind_locat.med_stay.nat+2*ind_locat.publ_tr.nat)/10 | [0,1] |
|  | ind_locat.all.reg | Individual location: all components (Regional) | (5*ind_locat.ind_mob.reg+3*ind_locat.med_stay.reg+2*ind_locat.publ_tr.reg)/10 | [0,1] |
|  | ind_locat.ind_mob.tot | Individual location: restricted individual mobility (Both levels) | max(ind_locat.ind_mob.nat,ind_locat.ind_mob.reg) | [0,1] |
|  | ind_locat.ind_mob.nat | Individual location: restricted individual mobility (National) | directly coded | [0,1] |
|  | ind_locat.ind_mob.reg | Individual location: restricted individual mobility (Regional) | directly coded | [0,1] |
|  | ind_locat.med_stay.tot | Individual location: conditional self-isolation (Both levels) | max(ind_locat.med_stay.nat,ind_locat.med_stay.reg) | [0,1] |
|  | ind_locat.med_stay.nat | Individual location: conditional self-isolation (National) | directly coded | [0,1] |
|  | ind_locat.med_stay.reg | Individual location: conditional self-isolation (Regional) | directly coded | [0,1] |
|  | ind_locat.publ_tr.tot | Individual location: closure of public transportation (Both levels) | max(ind_locat.publ_tr.nat,ind_locat.publ_tr.reg) | [0,1] |
|  | ind_locat.publ_tr.nat | Individual location: closure of public transportation (National) | directly coded | [0,1] |
|  | ind_locat.publ_tr.reg | Individual location: closure of public transportation (Regional) | directly coded | [0,1] |
| **Closure of places of congregation:** | | | | |
|  | places.all.tot | Closures of places of human congregation: all components (Both levels) | (2*places.gov_offs.tot+2*places.ne_busn.tot+2*places.restrts.tot+1*places.venues.tot+1*places.wfh.tot)/8 | [0,1] |
|  | places.all.nat | Closures of places of human congregation: all components (National) | (2*places.gov_offs.nat+2*places.ne_busn.nat+2*places.restrts.nat+1*places.venues.nat+1*places.wfh.nat)/8 | [0,1] |
|  | places.all.reg | Closures of places of human congregation: all components (Regional) | (2*places.gov_offs.reg+2*places.ne_busn.reg+2*places.restrts.reg+1*places.venues.reg+1*places.wfh.reg)/8 | [0,1] |
|  | places.gov_offs.tot | Closures of places of human congregation: closure of government offices (Both levels) | max(places.gov_offs.nat,places.gov_offs.reg) | [0,1] |
|  | places.gov_offs.nat | Closures of places of human congregation: closure of government offices (National) | directly coded | [0,1] |
|  | places.gov_offs.reg | Closures of places of human congregation: closure of government offices (Regional) | directly coded | [0,1] |
|  | places.ne_busn.tot | Closures of places of human congregation: closure of non-essential businesses (Both levels) | max(places.ne_busn.nat,places.ne_busn.reg) | [0,1] |
|  | places.ne_busn.nat | Closures of places of human congregation: closure of non-essential businesses (National) | directly coded | [0,1] |
|  | places.ne_busn.reg | Closures of places of human congregation: closure of non-essential businesses (Regional) | directly coded | [0,1] |
|  | places.restrts.tot | Closures of places of human congregation: closure of restaurants (Both levels) | max(places.restrts.nat,places.restrts.reg) | [0,1] |
|  | places.restrts.nat | Closures of places of human congregation: closure of restaurants (National) | directly coded | [0,1] |
|  | places.restrts.reg | Closures of places of human congregation: closure of restaurants (Regional) | directly coded | [0,1] |
|  | places.venues.tot | Closures of places of human congregation: closure of venues of entertainment and leisure (Both levels) | max(places.venues.nat,places.venues.reg) | [0,1] |
|  | places.venues.nat | Closures of places of human congregation: closure of venues of entertainment and leisure (National) | directly coded | [0,1] |
|  | places.venues.reg | Closures of places of human congregation: closure of venues of entertainment and leisure (Regional) | directly coded | [0,1] |
|  | places.wfh.tot | Closures of places of human congregation: working from home requirement (Both levels) | max(places.wfh.nat,places.wfh.reg) | [0,1] |
|  | places.wfh.nat | Closures of places of human congregation: working from home requirement (National) | directly coded | [0,1] |
|  | places.wfh.reg | Closures of places of human congregation: working from home requirement (Regional) | directly coded | [0,1] |
| **Schools and social gatherings:** | | | | |
|  | soc_and_schls.all.tot | Closure of schools and restrictions on social gatherings: all components (Both levels) | (4*soc_and_schls.schools.tot+4*soc_and_schls.soc_gath.tot)/8 | [0,1] |
|  | soc_and_schls.all.nat | Closure of schools and restrictions on social gatherings: all components (National) | (4*soc_and_schls.schools.nat+4*soc_and_schls.soc_gath.nat)/8 | [0,1] |
|  | soc_and_schls.all.reg | Closure of schools and restrictions on social gatherings: all components (Regional) | (4*soc_and_schls.schools.reg+4*soc_and_schls.soc_gath.reg)/8 | [0,1] |
|  | soc_and_schls.schools.tot | Closure of schools and restrictions on social gatherings: closure of schools (Both levels) | max(soc_and_schls.schools.nat,soc_and_schls.schools.reg) | [0,1] |
|  | soc_and_schls.schools.nat | Closure of schools and restrictions on social gatherings: closure of schools (National) | directly coded | [0,1] |
|  | soc_and_schls.schools.reg | Closure of schools and restrictions on social gatherings: closure of schools (Regional) | directly coded | [0,1] |
|  | soc_and_schls.soc_gath.tot | Closure of schools and restrictions on social gatherings: limits on size of social gatherings (Both levels) | max(soc_and_schls.soc_gath.nat,soc_and_schls.soc_gath.reg) | [0,1] |
|  | soc_and_schls.soc_gath.nat | Closure of schools and restrictions on social gatherings: limits on size of social gatherings (National) | directly coded | [0,1] |
|  | soc_and_schls.soc_gath.reg | Closure of schools and restrictions on social gatherings: limits on size of social gatherings (Regional) | directly coded | [0,1] |
| **Personal protective equipment:** | | | | |
|  | masks.all.tot | Mandatory wearing of personal protective equipment (Both levels) | max(masks.all.nat,masks.all.reg) | [0,1] |
|  | masks.all.nat | Mandatory wearing of personal protective equipment (National) | directly coded | [0,1] |
|  | masks.all.reg | Mandatory wearing of personal protective equipment (Regional) | directly coded | [0,1] |

**Country-level Protective Policy Index Method 2 (PPI-M2-N) dataset**

|  | Variable name | Description | Construction | Range |
| --- | --- | --- | --- | --- |
| **Case Identification variables:** | | | | |
|  | cname | Country name |  |  |
|  | isocode | ISO 3166 country code |  |  |
|  | isoabbr | country abbreviation according to ISO 3166 |  |  |
|  | date | date |  |  |
| **Protective Policy Index Method 2 (PPI-M2) calculated variables:** | | | | |
|  | ppi.all.tot.ave.2 | Protective Policy Index (Both levels) | Population-weighted average of region-specific values of ppi.all.tot.2 | [0,1] |
|  | ppi.all.nat.ave.2 | Protective Policy Index (National) | Population-weighted average of region-specific values of ppi.all.nat.2 | [0,1] |
|  | ppi.all.reg.ave.2 | Protective Policy Index (Regional) | Population-weighted average of region-specific values of ppi.all.reg.2 | [0,1] |
| **Borders:** | | | | |
|  | borders.all.tot.ave.2 | Border closures (Both levels) | Population-weighted average of region-specific values of borders.all.tot.2 | [0,1] |
|  | borders.all.nat.ave.2 | Border closures (National) | Population-weighted average of region-specific values of borders.all.nat.2 | [0,1] |
|  | borders.all.reg.ave.2 | Border closures (Regional) | Population-weighted average of region-specific values of borders.all.reg.2 | [0,1] |
| **State of emergency:** | | | | |
|  | emerg.all.tot.ave.2 | State of emergency (Both levels) | Population-weighted average of region-specific values of emerg.all.tot.2 | [0,1] |
|  | emerg.all.nat.ave.2 | State of emergency (National) | Population-weighted average of region-specific values of emerg.all.nat.2 | [0,1] |
|  | emerg.all.reg.ave.2 | State of emergency (Regional) | Population-weighted average of region-specific values of emerg.all.reg.2 | [0,1] |
| **Individual location restrictions:** | | | | |
|  | ind_locat.all.tot.ave.2 | Individual location: all components (Both levels) | Population-weighted average of region-specific values of ind_locat.all.tot.2 | [0,1] |
|  | ind_locat.all.nat.ave.2 | Individual location: all components (National) | Population-weighted average of region-specific values of ind_locat.all.nat.2 | [0,1] |
|  | ind_locat.all.reg.ave.2 | Individual location: all components (Regional) | Population-weighted average of region-specific values of ind_locat.all.reg.2 | [0,1] |
|  | ind_locat.ind_mob.tot.ave.2 | Individual location: restricted individual mobility (Both levels) | Population-weighted average of region-specific values of ind_locat.ind_mob.tot.2 | [0,1] |
|  | ind_locat.ind_mob.nat.ave.2 | Individual location: restricted individual mobility (National) | Population-weighted average of region-specific values of ind_locat.ind_mob.nat.2 | [0,1] |
|  | ind_locat.ind_mob.reg.ave.2 | Individual location: restricted individual mobility (Regional) | Population-weighted average of region-specific values of ind_locat.ind_mob.reg.2 | [0,1] |
|  | ind_locat.med_quar.tot.ave.2 | Individual location: mandatory quarantine (Both levels) | Population-weighted average of region-specific values of ind_locat.med_quar.tot.2 | [0,1] |
|  | ind_locat.med_quar.nat.ave.2 | Individual location: mandatory quarantine (National) | Population-weighted average of region-specific values of ind_locat.med_quar.nat.2 | [0,1] |
|  | ind_locat.med_quar.reg.ave.2 | Individual location: mandatory quarantine (Regional) | Population-weighted average of region-specific values of ind_locat.med_quar.reg.2 | [0,1] |
|  | ind_locat.med_stay.tot.ave.2 | Individual location: conditional self-isolation (Both levels) | Population-weighted average of region-specific values of ind_locat.med_stay.tot.2 | [0,1] |
|  | ind_locat.med_stay.nat.ave.2 | Individual location: conditional self-isolation (National) | Population-weighted average of region-specific values of ind_locat.med_stay.nat.2 | [0,1] |
|  | ind_locat.med_stay.reg.ave.2 | Individual location: conditional self-isolation (Regional) | Population-weighted average of region-specific values of ind_locat.med_stay.reg.2 | [0,1] |
|  | ind_locat.publ_tr.tot.ave.2 | Individual location: closure of public transportation (Both levels) | Population-weighted average of region-specific values of ind_locat.publ_tr.tot.2 | [0,1] |
|  | ind_locat.publ_tr.nat.ave.2 | Individual location: closure of public transportation (National) | Population-weighted average of region-specific values of ind_locat.publ_tr.nat.2 | [0,1] |
|  | ind_locat.publ_tr.reg.ave.2 | Individual location: closure of public transportation (Regional) | Population-weighted average of region-specific values of ind_locat.publ_tr.reg.2 | [0,1] |
| **Closure of places of congregation:** | | | | |
|  | places.all.tot.ave.2 | Closures of places of human congregation: all components (Both levels) | Population-weighted average of region-specific values of places.all.tot.2 | [0,1] |
|  | places.all.nat.ave.2 | Closures of places of human congregation: all components (National) | Population-weighted average of region-specific values of places.all.nat.2 | [0,1] |
|  | places.all.reg.ave.2 | Closures of places of human congregation: all components (Regional) | Population-weighted average of region-specific values of places.all.reg.2 | [0,1] |
|  | places.gov_offs.tot.ave.2 | Closures of places of human congregation: closure of government offices (Both levels) | Population-weighted average of region-specific values of places.gov_offs.tot.2 | [0,1] |
|  | places.gov_offs.nat.ave.2 | Closures of places of human congregation: closure of government offices (National) | Population-weighted average of region-specific values of places.gov_offs.nat.2 | [0,1] |
|  | places.gov_offs.reg.ave.2 | Closures of places of human congregation: closure of government offices (Regional) | Population-weighted average of region-specific values of places.gov_offs.reg.2 | [0,1] |
|  | places.ne_busn.tot.ave.2 | Closures of places of human congregation: closure of non-essential businesses (Both levels) | Population-weighted average of region-specific values of places.ne_busn.tot.2 | [0,1] |
|  | places.ne_busn.nat.ave.2 | Closures of places of human congregation: closure of non-essential businesses (National) | Population-weighted average of region-specific values of places.ne_busn.nat.2 | [0,1] |
|  | places.ne_busn.reg.ave.2 | Closures of places of human congregation: closure of non-essential businesses (Regional) | Population-weighted average of region-specific values of places.ne_busn.reg.2 | [0,1] |
|  | places.restrts.tot.ave.2 | Closures of places of human congregation: closure of restaurants (Both levels) | Population-weighted average of region-specific values of places.restrts.tot.2 | [0,1] |
|  | places.restrts.nat.ave.2 | Closures of places of human congregation: closure of restaurants (National) | Population-weighted average of region-specific values of places.restrts.nat.2 | [0,1] |
|  | places.restrts.reg.ave.2 | Closures of places of human congregation: closure of restaurants (Regional) | Population-weighted average of region-specific values of places.restrts.reg.2 | [0,1] |
|  | places.venues.tot.ave.2 | Closures of places of human congregation: closure of venues of entertainment and leisure (Both levels) | Population-weighted average of region-specific values of places.venues.tot.2 | [0,1] |
|  | places.venues.nat.ave.2 | Closures of places of human congregation: closure of venues of entertainment and leisure (National) | Population-weighted average of region-specific values of places.venues.nat.2 | [0,1] |
|  | places.venues.reg.ave.2 | Closures of places of human congregation: closure of venues of entertainment and leisure (Regional) | Population-weighted average of region-specific values of places.venues.reg.2 | [0,1] |
|  | places.wfh.tot.ave.2 | Closures of places of human congregation: working from home requirement (Both levels) | Population-weighted average of region-specific values of places.wfh.tot.2 | [0,1] |
|  | places.wfh.nat.ave.2 | Closures of places of human congregation: working from home requirement (National) | Population-weighted average of region-specific values of places.wfh.nat.2 | [0,1] |
|  | places.wfh.reg.ave.2 | Closures of places of human congregation: working from home requirement (Regional) | Population-weighted average of region-specific values of places.wfh.reg.2 | [0,1] |
| **Schools and social gatherings:** | | | | |
|  | soc_and_schls.all.tot.ave.2 | Closure of schools and restrictions on social gatherings: all components (Both levels) | Population-weighted average of region-specific values of soc_and_schls.all.tot.2 | [0,1] |
|  | soc_and_schls.all.nat.ave.2 | Closure of schools and restrictions on social gatherings: all components (National) | Population-weighted average of region-specific values of soc_and_schls.all.nat.2 | [0,1] |
|  | soc_and_schls.all.reg.ave.2 | Closure of schools and restrictions on social gatherings: all components (Regional) | Population-weighted average of region-specific values of soc_and_schls.all.reg.2 | [0,1] |
|  | soc_and_schls.schools.tot.ave.2 | Closure of schools and restrictions on social gatherings: closure of schools (Both levels) | Population-weighted average of region-specific values of soc_and_schls.schools.tot.2 | [0,1] |
|  | soc_and_schls.schools.nat.ave.2 | Closure of schools and restrictions on social gatherings: closure of schools (National) | Population-weighted average of region-specific values of soc_and_schls.schools.nat.2 | [0,1] |
|  | soc_and_schls.schools.reg.ave.2 | Closure of schools and restrictions on social gatherings: closure of schools (Regional) | Population-weighted average of region-specific values of soc_and_schls.schools.reg.2 | [0,1] |
|  | soc_and_schls.soc_gath.tot.ave.2 | Closure of schools and restrictions on social gatherings: limits on size of social gatherings (Both levels) | Population-weighted average of region-specific values of soc_and_schls.soc_gath.tot.2 | [0,1] |
|  | soc_and_schls.soc_gath.nat.ave.2 | Closure of schools and restrictions on social gatherings: limits on size of social gatherings (National) | Population-weighted average of region-specific values of soc_and_schls.soc_gath.nat.2 | [0,1] |
|  | soc_and_schls.soc_gath.reg.ave.2 | Closure of schools and restrictions on social gatherings: limits on size of social gatherings (Regional) | Population-weighted average of region-specific values of soc_and_schls.soc_gath.reg.2 | [0,1] |
| **Medical mandate:** | | | | |
|  | med_mandate.all.tot.ave.2 | Medical mandate: all components (Both levels) | Population-weighted average of region-specific values of med_mandate.all.tot.2 | [0,1] |
|  | med_mandate.all.nat.ave.2 | Medical mandate: all components (National) | Population-weighted average of region-specific values of med_mandate.all.nat.2 | [0,1] |
|  | med_mandate.all.reg.ave.2 | Medical mandate: all components (Regional) | Population-weighted average of region-specific values of med_mandate.all.reg.2 | [0,1] |
|  | med_mandate.dist_mand.tot.ave.2 | Medical mandate: social distancing mandates (Both levels) | Population-weighted average of region-specific values of med_mandate.dist_mand.tot.2 | [0,1] |
|  | med_mandate.dist_mand.nat.ave.2 | Medical mandate: social distancing mandates (National) | Population-weighted average of region-specific values of med_mandate.dist_mand.nat.2 | [0,1] |
|  | med_mandate.dist_mand.reg.ave.2 | Medical mandate: social distancing mandates (Regional) | Population-weighted average of region-specific values of med_mandate.dist_mand.reg.2 | [0,1] |
|  | med_mandate.masks.tot.ave.2 | Medical mandate: mandatory wearing of personal protective equipment (Both levels) | Population-weighted average of region-specific values of med_mandate.masks.tot.2 | [0,1] |
|  | med_mandate.masks.nat.ave.2 | Medical mandate: mandatory wearing of personal protective equipment (National) | Population-weighted average of region-specific values of med_mandate.masks.nat.2 | [0,1] |
|  | med_mandate.masks.reg.ave.2 | Medical mandate: mandatory wearing of personal protective equipment (Regional) | Population-weighted average of region-specific values of med_mandate.masks.reg.2 | [0,1] |

**Sub-national level Protective Policy Indices by Method 2 (PPI-M2-R)**

|  | Variable name | Description | Construction | Range |
| --- | --- | --- | --- | --- |
| **Case Identification variables:** | | | | |
|  | cname | Country name |  |  |
|  | isocode | ISO 3166 country code |  |  |
|  | isoabbr | country abbreviation according to ISO 3166 |  |  |
|  | state_province | the name of the administrative division where the policy applies |  |  |
|  | iso_state | ISO 3166 code of the administrative division where the policy applies |  |  |
|  | date | date |  |  |
| **Protective Policy Index Method 1 (PPI-M2) calculated variables:** | | | | |
|  | ppi.all.tot.2 | Protective Policy Index (Both levels) | (8*borders.all.tot.2+3*emerg.all.tot.2+20*ind_locat.all.tot.2+16*places.all.tot.2+16*soc_and_schls.all.tot.2+10*med_mandate.all.tot.2)/73 | [0,1] |
|  | ppi.all.nat.2 | Protective Policy Index (National) | (8*borders.all.nat.2+3*emerg.all.nat.2+20*ind_locat.all.nat.2+16*places.all.nat.2+16*soc_and_schls.all.nat.2+10*med_mandate.all.nat.2)/73 | [0,1] |
|  | ppi.all.reg.2 | Protective Policy Index (Regional) | (8*borders.all.reg.2+3*emerg.all.reg.2+20*ind_locat.all.reg.2+16*places.all.reg.2+16*soc_and_schls.all.reg.2+10*med_mandate.all.reg.2)/73 | [0,1] |
| **Borders:** | | | | |
|  | borders.all.tot.2 | Border closures (Both levels) | max(borders.all.nat.2,borders.all.reg.2) | [0,1] |
|  | borders.all.nat.2 | Border closures (National) | max(borders.all.nat,borders.all.nat) | [0,1] |
|  | borders.all.reg.2 | Border closures (Regional) | max(borders.all.reg,borders.all.reg) | [0,1] |
| **State of emergency:** | | | | |
|  | emerg.all.tot.2 | State of emergency (Both levels) | max(emerg.all.nat.2,emerg.all.reg.2) | [0,1] |
|  | emerg.all.nat.2 | State of emergency (National) | directly coded | [0,1] |
|  | emerg.all.reg.2 | State of emergency (Regional) | directly coded | [0,1] |
| **Individual location restrictions:** | | | | |
|  | ind_locat.all.tot.2 | Individual location: all components (Both levels) | (10*ind_locat.ind_mob.tot.2+4*ind_locat.med_quar.tot.2+2*ind_locat.med_stay.tot.2+4*ind_locat.publ_tr.tot.2)/20 | [0,1] |
|  | ind_locat.all.nat.2 | Individual location: all components (National) | (10*ind_locat.ind_mob.nat.2+4*ind_locat.med_quar.nat.2+2*ind_locat.med_stay.nat.2+4*ind_locat.publ_tr.nat.2)/20 | [0,1] |
|  | ind_locat.all.reg.2 | Individual location: all components (Regional) | (10*ind_locat.ind_mob.reg.2+4*ind_locat.med_quar.reg.2+2*ind_locat.med_stay.reg.2+4*ind_locat.publ_tr.reg.2)/20 | [0,1] |
|  | ind_locat.ind_mob.tot.2 | Individual location: restricted individual mobility (Both levels) | max(ind_locat.ind_mob.nat.2,ind_locat.ind_mob.reg.2) | [0,1] |
|  | ind_locat.ind_mob.nat.2 | Individual location: restricted individual mobility (National) | directly coded | [0,1] |
|  | ind_locat.ind_mob.reg.2 | Individual location: restricted individual mobility (Regional) | directly coded | [0,1] |
|  | ind_locat.med_quar.tot.2 | Individual location: mandatory quarantine (Both levels) | max(ind_locat.med_quar.nat.2,ind_locat.med_quar.reg.2) | [0,1] |
|  | ind_locat.med_quar.nat.2 | Individual location: mandatory quarantine (National) | directly coded | [0,1] |
|  | ind_locat.med_quar.reg.2 | Individual location: mandatory quarantine (Regional) | directly coded | [0,1] |
|  | ind_locat.med_stay.tot.2 | Individual location: conditional self-isolation (Both levels) | max(ind_locat.med_stay.nat.2,ind_locat.med_stay.reg.2) | [0,1] |
|  | ind_locat.med_stay.nat.2 | Individual location: conditional self-isolation (National) | directly coded | [0,1] |
|  | ind_locat.med_stay.reg.2 | Individual location: conditional self-isolation (Regional) | directly coded | [0,1] |
|  | ind_locat.publ_tr.tot.2 | Individual location: closure of public transportation (Both levels) | max(ind_locat.publ_tr.nat.2,ind_locat.publ_tr.reg.2) | [0,1] |
|  | ind_locat.publ_tr.nat.2 | Individual location: closure of public transportation (National) | directly coded | [0,1] |
|  | ind_locat.publ_tr.reg.2 | Individual location: closure of public transportation (Regional) | directly coded | [0,1] |
| **Closure of places of congregation:** | | | | |
|  | places.all.tot.2 | Closures of places of human congregation: all components (Both levels) | (4*places.gov_offs.tot.2+4*places.ne_busn.tot.2+4*places.restrts.tot.2+2*places.venues.tot.2+2*places.wfh.tot.2)/16 | [0,1] |
|  | places.all.nat.2 | Closures of places of human congregation: all components (National) | (4*places.gov_offs.nat.2+4*places.ne_busn.nat.2+4*places.restrts.nat.2+2*places.venues.nat.2+2*places.wfh.nat.2)/16 | [0,1] |
|  | places.all.reg.2 | Closures of places of human congregation: all components (Regional) | (4*places.gov_offs.reg.2+4*places.ne_busn.reg.2+4*places.restrts.reg.2+2*places.venues.reg.2+2*places.wfh.reg.2)/16 | [0,1] |
|  | places.gov_offs.tot.2 | Closures of places of human congregation: closure of government offices (Both levels) | max(places.gov_offs.nat.2,places.gov_offs.reg.2) | [0,1] |
|  | places.gov_offs.nat.2 | Closures of places of human congregation: closure of government offices (National) | directly coded | [0,1] |
|  | places.gov_offs.reg.2 | Closures of places of human congregation: closure of government offices (Regional) | directly coded | [0,1] |
|  | places.ne_busn.tot.2 | Closures of places of human congregation: closure of non-essential businesses (Both levels) | max(places.ne_busn.nat.2,places.ne_busn.reg.2) | [0,1] |
|  | places.ne_busn.nat.2 | Closures of places of human congregation: closure of non-essential businesses (National) | directly coded | [0,1] |
|  | places.ne_busn.reg.2 | Closures of places of human congregation: closure of non-essential businesses (Regional) | directly coded | [0,1] |
|  | places.restrts.tot.2 | Closures of places of human congregation: closure of restaurants (Both levels) | max(places.restrts.nat.2,places.restrts.reg.2) | [0,1] |
|  | places.restrts.nat.2 | Closures of places of human congregation: closure of restaurants (National) | directly coded | [0,1] |
|  | places.restrts.reg.2 | Closures of places of human congregation: closure of restaurants (Regional) | directly coded | [0,1] |
|  | places.venues.tot.2 | Closures of places of human congregation: closure of venues of entertainment and leisure (Both levels) | max(places.venues.nat.2,places.venues.reg.2) | [0,1] |
|  | places.venues.nat.2 | Closures of places of human congregation: closure of venues of entertainment and leisure (National) | directly coded | [0,1] |
|  | places.venues.reg.2 | Closures of places of human congregation: closure of venues of entertainment and leisure (Regional) | directly coded | [0,1] |
|  | places.wfh.tot.2 | Closures of places of human congregation: working from home requirement (Both levels) | max(places.wfh.nat.2,places.wfh.reg.2) | [0,1] |
|  | places.wfh.nat.2 | Closures of places of human congregation: working from home requirement (National) | directly coded | [0,1] |
|  | places.wfh.reg.2 | Closures of places of human congregation: working from home requirement (Regional) | directly coded | [0,1] |
| **Schools and social gatherings:** | | | | |
|  | soc_and_schls.all.tot.2 | Closure of schools and restrictions on social gatherings: all components (Both levels) | (8*soc_and_schls.schools.tot.2+8*soc_and_schls.soc_gath.tot.2)/16 | [0,1] |
|  | soc_and_schls.all.nat.2 | Closure of schools and restrictions on social gatherings: all components (National) | (8*soc_and_schls.schools.nat.2+8*soc_and_schls.soc_gath.nat.2)/16 | [0,1] |
|  | soc_and_schls.all.reg.2 | Closure of schools and restrictions on social gatherings: all components (Regional) | (8*soc_and_schls.schools.reg.2+8*soc_and_schls.soc_gath.reg.2)/16 | [0,1] |
|  | soc_and_schls.schools.tot.2 | Closure of schools and restrictions on social gatherings: closure of schools (Both levels) | max(soc_and_schls.schools.nat.2,soc_and_schls.schools.reg.2) | [0,1] |
|  | soc_and_schls.schools.nat.2 | Closure of schools and restrictions on social gatherings: closure of schools (National) | directly coded | [0,1] |
|  | soc_and_schls.schools.reg.2 | Closure of schools and restrictions on social gatherings: closure of schools (Regional) | directly coded | [0,1] |
|  | soc_and_schls.soc_gath.tot.2 | Closure of schools and restrictions on social gatherings: limits on size of social gatherings (Both levels) | max(soc_and_schls.soc_gath.nat.2,soc_and_schls.soc_gath.reg.2) | [0,1] |
|  | soc_and_schls.soc_gath.nat.2 | Closure of schools and restrictions on social gatherings: limits on size of social gatherings (National) | directly coded | [0,1] |
|  | soc_and_schls.soc_gath.reg.2 | Closure of schools and restrictions on social gatherings: limits on size of social gatherings (Regional) | directly coded | [0,1] |
| **Medical mandate:** | | | | |
|  | med_mandate.all.tot.2 | Medical mandate: all components (Both levels) | (4*med_mandate.dist_mand.tot.2+6*med_mandate.masks.tot.2)/10 | [0,1] |
|  | med_mandate.all.nat.2 | Medical mandate: all components (National) | (4*med_mandate.dist_mand.nat.2+6*med_mandate.masks.nat.2)/10 | [0,1] |
|  | med_mandate.all.reg.2 | Medical mandate: all components (Regional) | (4*med_mandate.dist_mand.reg.2+6*med_mandate.masks.reg.2)/10 | [0,1] |
|  | med_mandate.dist_mand.tot.2 | Medical mandate: social distancing mandates (Both levels) | max(med_mandate.dist_mand.nat.2,med_mandate.dist_mand.reg.2) | [0,1] |
|  | med_mandate.dist_mand.nat.2 | Medical mandate: social distancing mandates (National) | directly coded | [0,1] |
|  | med_mandate.dist_mand.reg.2 | Medical mandate: social distancing mandates (Regional) | directly coded | [0,1] |
|  | med_mandate.masks.tot.2 | Medical mandate: mandatory wearing of personal protective equipment (Both levels) | max(med_mandate.masks.nat.2,med_mandate.masks.reg.2) | [0,1] |
|  | med_mandate.masks.nat.2 | Medical mandate: mandatory wearing of personal protective equipment (National) | directly coded | [0,1] |
|  | med_mandate.masks.reg.2 | Medical mandate: mandatory wearing of personal protective equipment (Regional) | directly coded | [0,1] |

**Event file (Discrete Policy Announcements)**

**changes_regions_m1**: Stores information about the reports that brought about the changes in the active policies included in PPI_regions_ZZ_m1 files

|  | Variable name | Description | Construction | Range |
| --- | --- | --- | --- | --- |
| **Case Identification variables:** | | | | |
|  | cname | Country name |  |  |
|  | isocode | ISO 3166 country code |  |  |
|  | isoabbr | country abbreviation according to ISO 3166 |  |  |
|  | state_province | the name of the administrative division where the policy applies |  |  |
|  | iso_state | ISO 3166 code of the administrative division where the policy applies |  |  |
|  | date | date |  |  |
|  | dimension | policy dimension |  | {air_bord, land_bord, sea_bord, soc_gath, schools, emerg, venues, restrts, ne_busn, gov_offs, ind_mob, med_stay, wfh, publ_tr, masks} |
|  | subnational | binary indicator marking whether the change applies to the national or regional policies |  | {0,1} |
| **Calculated case variables:** | | | | |
|  | total_change | total change in the policy value from the preceding date | difference between the policy values on the given dimension in a given locale on the current date from the preceding date | [-1,1] |
| **Report variables:** | | | | |
|  | branch | branch of government announcing the policy |  | {executive leadership, bureaucracy, legislative, judiciary, missing, other} |
|  | who | contains information on who announced the policy |  |  |
|  | institution | contains information on what institution announced the policy |  |  |
|  | report_date | the date of the report used to code the policy/policy announcement date |  |  |
|  | exportation_date | the date when the policy expires |  |  |
|  | report_change | the change in the policy value from the preceding date according to the report |  |  |
